# Ecbolic Action of Pilocarpin

**Published:** 1885-07

**Authors:** A. Lagorio


ECBOLIC ACTION OF PILOCARPIN.

The important action of pilocarpin in the production of premature labour has already been recognized by some writers, and a few cases have been placed on record. Dr E. Quintavalle has recently had an opportunity to test this action, and obtain a most satisfactory result. The case, presenting many points of interest, has been published in the March number of *Il Morgagni.*

The patient was 23 years old, married two years and a primipara. When a girl she had suffered with acute articular rheumatism; menstruated in the 15th year; and had an abortion three months after marriage. Since the commencement of her last pregnancy she has suffered constantly from great dyspnœa. She never consulted any physician, till now, when being in the 7th month of her pregnancy, and her sufferings becoming greater, she decided to do so. Dr. Quintavalle found her in sitting posture in bed, any other position being impossible for her, presenting marked dyspnœa and tumultuous pulsations of the heart. No physical examinations could be made by percussion nor with a stethoscope, for the patient could not bear the slightest pressure on the thoracic walls. Her face was swollen of a dark-red color; the lower limbs and hands were œdematous; the belly was slightly larger than the usual proportions observed in the 7th month of pregnancy. Fatigued by the frequency and shortness of breath, the voice did not rise above a low whisper; she urinated very little, and none of the urine being present, the doctor could not examine it. Unable to make any accurate examination, Quintavalle, taking in account the past history of the patient, made a diagnosis of an acute stenosis of the left auriculo-ventricular orifice, due to an endocarditis developed during the pregnancy, and recognized in the marked pulmonary stasis the actual fact most dangerous to the life of the patient. To treat the stasis he bled the patient, which produced a slight improvement. He administered digitalis for the heart-trouble, and did not omit cutaneous derivatives. He called again to see her three weeks after, in company with another practitioner, and found the patient much worse. He thought it prudent not to use the same therapeutic means as before, but decided to proceed to induce premature labour. Nothing else remained to be done but to select the most suitable method. The condition of the patient did not permit the use of the ordinary means. Remembering the good effects of pilocarpin, both agreed to try its value. Six centigrams (1 grain) were prescribed dissolved in three grains of water, and a third part was immediately injected hypodermically into the internal part of the left thigh. After eight or ten minutes the physiological action of jaborandi was observed: mydriasis, profuse salivation and perspiration with nausea. A digital examination made after 15 minutes showed no change in the uterine neck. The general phenomena being now almost dissipated, another injection of 2 centigrams was made in the right thigh. The same physiological action of the remedy reappeared, together with a relative euphoria in the conditions of the patient. At the same time she began to notice some sensations which showed a beginning of uterine contractions. After waiting twenty minutes and these not increasing, two centigrams more of the alkaloid were injected: the same result followed its use, attended by a greater freedom of respiration and a notable diminution in the symptoms of stasis. The womb now began to act. The patient was left under the care of a midwife and was safely delivered, after 30 hours from the first injection of pilocarpin, of a vigorous, sound and living child.

Having ascertained this effect of muriate of pilocarpin in the induction of premature labour, it remains to be proven in what way jaborandi can produce this result. There is a physiological reason which permits us to give an explanation, plausible at least, of the ecbolic action of pilocarpin. It is known that where pilocarpin is introduced into the system hypodermically, it immediately produces a diminished pressure of the arterial system : this can be recognized by looking at the profuse salivation and perspiration, which phenomena are really the expression of a sudden nervous disorder, which exactly agrees with the fact of a temporary anæmia of the centers of innervation. This anæmia of the nervous centers produces different forms of neuroses, which we observe daily. These neuroses are centric or periphereral, circumscribed or diffused, according to the seat of the anæmia and according to its completeness.

Then we can see how pilocarpin can produce premature labour by diminishing the arterial pressure, producing therefore an anæmia of the center innervation of the uterus, and causing a sudden neurosis of this organ, greatly predisposed to it by its state of gestation.

Quintavalle recommends the use of hypodermic injections of pilocarpin to induce premature labour, for it improves the chances of both mother and child ; and we know the dangers of the ordinary processes, and the great repugnance to their application. A. LAGORIO.

